# Characterisation of human exposure to nocturnal biting by malaria and arbovirus vectors in a rural community in Chókwè district, southern Mozambique

**DOI:** 10.12688/wellcomeopenres.19278.1

**Published:** 2023-05-02

**Authors:** Ayubo Kampango, João Pinto, Ana Paula Abílio, Elias Machoe, Júlio Matusse, Philip J. McCall

**Affiliations:** 1 Sector de Estudo de Vectores, Instituto Nacional de Saúde (INS), Maputo, Villa de Marracuene EN1, Plot 3943, Mozambique; 2 Department of Zoology and Entomology, University of Pretoria, Pretoria, Hatfield, 0028, South Africa; 3 Global Health and Tropical Medicine, Institute of Hygiene and Tropical Medicine (IHMT), Lisbon, Rua da Junqueira, 100 1349-008, Portugal; 4 Vector Biology Department, Liverpool School of Tropical Medicine (LSTM), Liverpool, Pembroke Place, L3 5QA, UK

**Keywords:** Malaria, arboviruses, mosquito vectors, exophagy, endophily, man-biting exposure, Massavasse village, Mozambique

## Abstract

**Background:** Understanding the magnitude of human exposure to mosquito biting is fundamental to reduce pathogen transmission. Here we report on a study quantifying the levels of mosquitoes attacking humans throughout the night in a rural area of Southern Mozambique.

**Methods:** Surveys were carried out in Massavasse village, southern Mozambique. The abundance and composition of host-seeking mosquito communities at night were assessed by human-landing catches (HLC) at one-hour intervals. Periods when people were located predominantly outdoors or indoors were used to estimate the amount of residents’ exposure to mosquito bites in either location, to explore the potential impact a bed net could have had in reducing biting by each vector species.

**Results:** A total of 69,758 host-seeking female mosquitoes comprising 23 species in four genera were collected. The exposure to biting by virtually all vector species was consistently high outdoors, typically at early evening and morning, with exception of *An. gambiae* *s.l* which was likely of biting a person with nearly same intensity indoors and outdoors throughout the night. Bed nets use could have reduced biting by *An. gambiae s.l* (dominated by *An. arabiensis*), *Ma. africana*, *Ma. uniformis*, *Cx. pipiens*, *Cx. antennatus*, and *Cx. poicilipes* by 53%, 47%, 46%, 38%, 31%, and 28% respectively, compared to non-users. Conversely, a bed net user would have had little protection against *An. pharoensis*, *An. ziemanni*, *An. tenebrosus*, and *Cx. tritaeniorhynchus* biting exposures.

**Conclusions:** This study showed that Massavasse residents were exposed to high levels of outdoor biting by malaria and arbovirus vectors that abound in the village. The findings help to identify entomological drivers of persistent malaria transmission in Mozambique and identify a wide range of arbovirus vectors nocturnally active in rural areas, many with outbreak potential. The study highlights the need for a surveillance system for monitoring arboviral diseases vectors in Mozambique.

## Introduction

Mosquitoes are responsible for transmitting some of the deadliest and most debilitating diseases to humans. Today, more than half of the global population is at risk from mosquito-borne diseases such as malaria, dengue, chikungunya, yellow fever, Japanese encephalitis and West Nile fever ^1^. These diseases have caused enormous suffering and remain a lasting impediment to societal development of endemic countries, particularly those in sub-Saharan Africa ^2^. Collectively, mosquito transmitted arboviral diseases, notably, dengue, chikungunya, Japanese encephalitis, and West Nile fever have been steadily increasing in geographic range, and in the frequency and magnitude of outbreaks ^3– 6^. Dengue incidence has increased more than 30 fold over the last three decades, becoming one of the world’s most common and rapidly widespread arboviral disease primarily, but not only, due to the expansion of its main vectors ^3, 7– 10^.

Historical and recent evidence indicate that it is highly likely that Mozambique is endemic for at least some arboviruses, most notably dengue, chikungunya, and Rift-valley fever ^11– 15^. However, the burden and geographic distribution of these and other arboviral diseases remain largely unknown as are the identities of the vectors apart from the major species. 

Mozambique accounts for over 6% of global malaria deaths and 4.1% of malaria cases ^16^, and the high rate of malaria transmission is the basis for classification of malaria as a major public health threat in Mozambique. While undoubtedly true, this has also served to divert attention away from other important vector-borne diseases. For example, a recent study reported that in 22 health units across 11 provinces of Mozambique, nearly 72% of febrile cases classed as malaria-negative had received treatment for malaria ^17^, without consideration of other possible causes, including arbovirus infections. However, the recent dengue outbreaks in the northern provinces of Nampula and Cabo Delgado ^18^ have come as a sharp reminder of the presence of mosquito-borne diseases other than malaria in many areas of Mozambique and of the need for even basic studies on the vectors ecology.

More than a hundred mosquito species have been recorded in Mozambique, including many known malaria and arbovirus vector species ^19– 22^. However, the public health importance of local mosquito fauna remains poorly determined, though a great proportion of them have been found infected with pathogenic viruses ^21^. The historic interest in malaria and the focus on controlling transmission is the most likely explanation why most entomological studies focused on the main malaria vectors in the *Anopheles gambiae* complex and the *An. funestus* group, neglecting the possibility of secondary malaria vectors or the primary arbovirus vectors occurring in the same areas.

The diversity of mosquito vector species reported from Mozambique suggests complexity in the transmission ecology of mosquito-borne diseases with unknown, yet potentially imminent, risks of outbreaks. Effective vaccines are few, and vector control interventions remain the most reliable and cost-effective control measures against virtually all known mosquito-borne arboviruses ^23^. Therefore, successful elimination or control of a vector relies on understanding the dynamics of its behaviour and, variations of key transmission indicators, notably the incidence of mosquito contact with a human, also known as the human-biting rate (HBR) ^24, 25^. When calculated correctly, the HBR gives the most realistic estimation of the magnitude of human exposure to a mosquito vector over time and space and, in combination with information on the human host‘s nocturnal and diurnal behavioural habits, it can indicate the likely success of protection methods directed at individuals or the community ^26, 27^. Apart from information on the dominant malaria vectors *An. funestus* and *An. arabiensis* in a few regions ^28– 32^, detailed patterns of nightly human exposure to host-seeking mosquito vectors in Mozambique is scant. The timing and diurnal cycles of vector activity are dynamic, often changing according to geographic regions ^26, 33^, local environmental and climatic conditions ^28, 34, 35^, host availability ^36^, or vector control pressure ^37– 39^.

In-depth and site-specific characterization of human exposure to mosquitoes is essential when designing and deploying accurate vector control to optimally target host habits. As such, the overreaching goal of this study was to investigate patterns of human exposure to potential vectors of malaria and arboviruses in Massavasse village, an irrigated rice ecosystem located in the Chókwè district in southern Mozambique.

## Methods

### Study site

**Figure 1.**
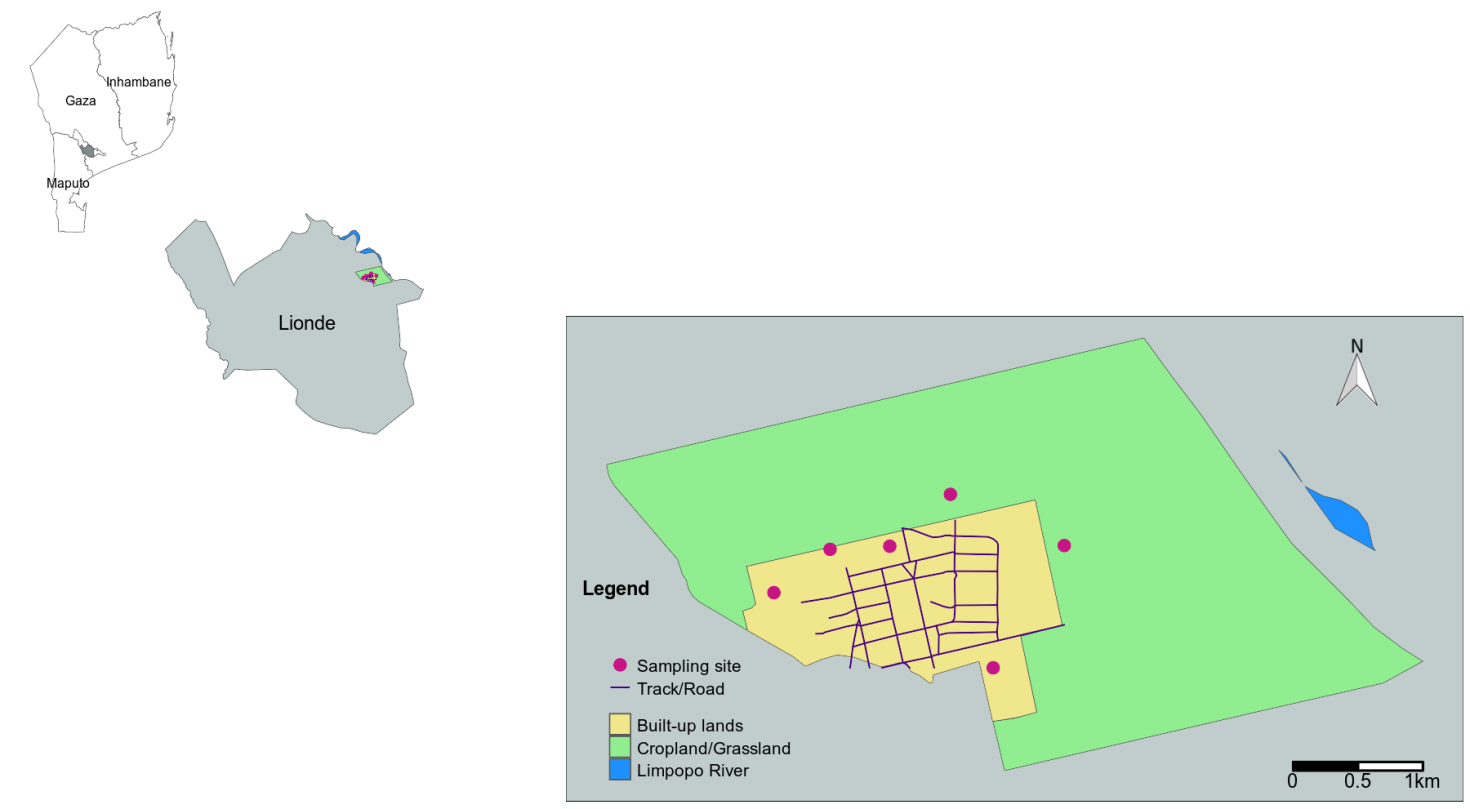
Map of south Mozambique, showing the location of area studied at Massavasse village in the Lionde administrative post, Chókwè district, Gaza province. Mozambique administrative border polygons and roads, and the polygons of river lines and water areas were obtained from the Humanitarian Data Exchange ( https://data.humdata.org/). Polygons of cropland areas around Massavasse village were made by authors with Google Earth Pro, using global croplands data obtained from the Global Land Analysis & Discovery ( https://glad.umd.edu/dataset/croplands?s=03).

Entomological surveys were carried out in 2016 in Massavasse village (-24.624839 ^o^S; 33.111787 ^o^E). Massavasse village is in Lionde Administrative Post, southeast Gaza province, southern Mozambique ( Figure 1). The village has one of the highest malaria prevalence compared to other regions in southern Mozambique, and a well characterized mosquito population ^31^. It comprises one of the most intensively irrigated areas of Mozambique ( Figure 1). The hot and rainy season in the village ranges from October to April, whereas the dry and cold season ranges from May to September. The mean air temperature during the hot/rainy season ranges between 25°C and 34°C, and 22°C and 16°C during the winter/dry season; the maximum average annual rainfall is 600 mm. The village has been inhabited by at least 4,711 individuals divided in, at least 989 households ^40^. The villagers grow rice in the irrigated fields surrounding the village, and at the lowlands of Limpopo River, which delineates the village from north to east ( Figure 1). Four main irrigations channels bordering the village provide temporary and permanent breeding sites for many mosquito species all year round. Known malaria vector species include members of *Anopheles gambiae* complex and *An. funestus* group and several other species including *An. pharoensis*, *An. tenebrosus*, *An. ziemanni*. More than 15 species of culicines, including known arbovirus vectors, such as *Culex tritaeniorhynchus*, *Cx. poicilipes*, *Cx. Antennatus*. *Mansonia uniformis*, *Man. africana*, *Aedes sudanensis*, among others, have also been found in the village.

### Mosquito collection

**Figure 2.**
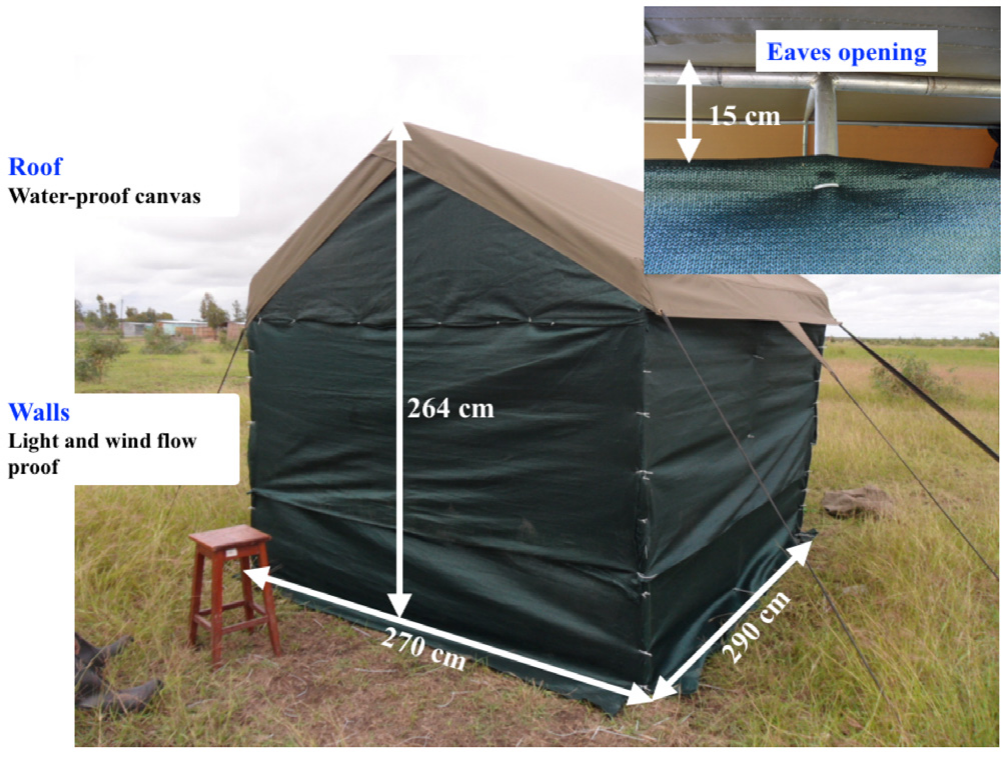
Details of the experimental hut used for indoor host-seeking mosquito collections.

Mosquito surveys were carried out from February to April 2016. Paired indoor and outdoor human landing catches were conducted in neighbourhoods two to six of Massavasse village ( Figure 1). In each neighbourhood, on each sampling date, two sampling locations were randomly selected and two purpose-built sentinel huts ( Figure 2) were placed at least fifty metres apart at each location. Mosquito collections were conducted hourly from 19:00 to 05:00 with pairs of collectors, one pair seated inside and other outside the sentinel huts, with legs uncovered. Mosquitoes were collected after alighting but before biting the collectors’ legs. Collectors were randomly assigned to the sentinel hut positions and undertook landing collections for a period of one hour after which they were rotated between positions in subsequent collection periods, allowing collectors to alternate between sentinel points and allow one hour’s rest between collections periods avoiding unnecessary interruptions and reducing the potential influence of relative attractiveness ^41^ and collector fatigue on mosquito catches. The time of sunset and sunrise was obtained with a hand-held GPS unit (Garmin e-Trex ^®^ H; Garmin Ltd, Southampton, U.K.).

### Description of sentinel huts

The portable sentinel huts were built from polyethylene shade cloth walls on aluminium frames measuring 290 cm x 264 cm x 270 cm ( Figure 2). The roof was made of 100% waterproof canvas. The walls had no windows and were composed of a high-density polyethylene shade cloth locally obtained, that purportedly blocks 98% of ultraviolet rays and 95% of visible light ( manufacturer’s claim). Previously, the same material had been shown to prevent 99% of indoor draughts or air currents ^42^. On this basis, we assumed that the only possible route for the cues from the hut interior would be through the 15 cm ‘eave’ apertures between the walls and the roof (Figure 2S). The walls were fastened to the frame with plastic cable ties to facilitate rapid assembly and deconstruction. When complete, the roof was secured with guy lines, to minimise movement. During periods when it was in use, the average temperature inside the hut was 27°C (range 25°C - 28°C).

### Sleep survey

The start and end times of sleep periods and the time spent indoors and outdoors by inhabitants were determined from data collected at 312 of the 989 households that existed in Massavasse village at the time. At least 60 to 62 randomly chosen households were visited in each of the five neighbourhoods studied. A household was defined as a house or compound occupied by a group of individuals during the study. Only adult individuals (≥ 18 years old) were interviewed based on their willingness to participate. Permission to interview the participate was sought from the head of the household. Everybody that accepted to be interviewed, including the head of the household, signed an informed consent form.

Participants were administrated a questionnaire containing questions to understand where they slept at night, what time they slept at night and woke up in the morning. In those cases where a household had more than one adult, individuals were interviewed separately to prevent them from influencing each other in their responses.

The survey was undertaken from afternoon to near sunset when most of the people were expected to have returned home from their daily duties. A copy of the questionnaire used can be found in Extended data.

### Sample processing and analysis

Collected mosquito samples were transported to the insectary and euthanized by putting paper cups with specimens inside a refrigerator for 20 to 30 minutes. Female and male *Anopheles* mosquitoes were morphologically identified to species according to taxonomic keys from Gillies & De Meillon ^43^ and Gillies & Coetzee ^44^, whereas non- *Anopheles* mosquitoes were identified using taxonomic keys proposed by Edwards ^45^; Jupp ^46^; Harbach ^47^ and Service ^48^. Mosquito samples that could not be morphologically identified, such as sibling species members of *An. gambiae* complex and *An*. *funestus* group, were further identified by molecular analysis (PCR) using the protocols proposed by Scott *et al*., ^49^ and Koekemoer *et al*., ^50^, respectively.

## Data analysis

### Estimation of human-biting rates

Estimation of human exposure to mosquito bites was only carried out for species where the total catch was equal to or greater than 1% of all mosquitoes caught.

Crude hourly mosquito biting density was determined as the average number of mosquitoes landing on a person/site/night. The hourly man-biting density of a given mosquito species was expressed in terms of Williams’s mean (Mw), described as follows: ${\text{M}}_{\text{w}}=exp\left(\frac{\sum ln(n_{i}+1)}{N}\right)-1$ , where *n _i_* is the total number of species *i,* and *N* is the number of sampling days. The Williams´ mean is a more appropriate measure of central tendency than the arithmetic mean, since the estimate is less affected by unusually small or very large samples ^51^, not an uncommon event when sampling mosquito populations ^52^.

Behaviour-adjusted hourly total number of bites experienced by unprotected individuals ( *B _u_*) were calculated based on data from both human and mosquito behaviours, as suggested by Killeen *et al* ^27^. As such, the biting rate of a mosquito species *i*, experienced by unprotected individuals at time ( *t*) was estimated based on the proportion of people reported to have stayed indoors ( *S _t_*) multiplied by crude indoor biting rate by species *i* at time *t* ( *B _Iit_*) plus the proportion of people reported to have stayed outdoors ( *1-S _t_*) multiplied by the crude outdoor biting rate ( *B _Oit_*):


$$B_{uit}=B_{Iit}S_{t}+B_{Oit}(1-S_{t})\;(1)$$

Thus, the overall nightly biting rate by mosquito species *i* experienced by an unprotected individual was calculated by summing biting rates for each observation hour, that is:


$$B_{ui}=\sum_{t=1}^{n}B_{I,t}S_{t}+B_{O,t}(1-S_{t})\;(2)$$

Where t = 1 corresponded to period from 19:00 – 20:00, t = 2 period 20:00 – 21:00, and continues up to time t = n, which in our case corresponded to period from 05:00 hrs – 06:00. We defined an unprotected individual as someone lacking any type of insecticide treated bed nets (ITNs). The biting exposure by species *i* at time *t* for a potentially protected individual was estimated by combining mosquito biting rate over time ( *t*), the sleeping behaviour of humans, and the protective efficacy of ITNs ( *P*), assumed to be constant, which was, as in Killeen *et al.*, ^27^. The effective adherence of ITNs use at a given time of the night was assumed to be equivalent to the proportion of people sleeping at that time ( *S _t_*).

At the time of writing, there have been no investigations in Mozambique of how human habits and behaviours might compromise or enhance the protective efficacy of insecticide treated nets ITNs). Hence, we explored the extent to which an ITN would potentially protect an individual human against mosquito bites, assuming a conservative average minimum protective efficacy level of 80% ( *P* = 0.8) for ITNs, based on studies conducted elsewhere in Africa ^53– 56^. When an individual is sleeping under an ITN, this is equivalent to a relative exposure to bites of 20% (i.e., 100 – 80) ^27^. The biting exposure experienced by protected individuals ( *Bp*) was estimated, according to Killeen *et al.*, ^27^ as follows:


$$B_{pit}=B_{Iit}S_{t}(1-P)+B_{Oit}(1-S_{t})\;(3)$$

Residents were assumed to have remained outdoors after emerging from indoor in the morning, and thus to have been exposed to bites by mosquitoes seeking hosts outdoors. Residents’ sleeping hours were assumed to be spent indoors and under an ITN.

The proportion of mosquito bites experienced indoors for an unprotected individual ( *π _I_*) was estimated by dividing the total number of bites received indoors ( *B _I_*) by the total number of bites received by unprotected individuals, as follows:


$$\pi_{Iit}=\frac{B_{Iit}S_{t}}{B_{Iit}S_{t}+B_{Oit}(1-S_{t})}\;(4)$$

The proportion of bites experienced outdoors for an unprotected human ( *π _O_*) was estimated as π _*Oit*_ = 1 - *π _Iit_*. Similarly, the relative proportion of biting for protected ( *π _pi_*) individual was estimated as:


$${\text{π}}_{pi}=\frac{B_{pit}}{B_{uit}}=\frac{B_{Iit}S_{t}(1-P)+B_{Oit}(1-S_{t})}{B_{Iit}S_{t}+B_{Oit}(1-S_{t})}\;(5)$$

### Statistical analysis

The significance of differences in magnitude between indoor bite exposure vs outdoor bite exposure was estimated using generalized linear models and assuming negative binomial distribution of mosquito counts with log link to explanatory variables. Response variables were mosquito counts, and predictor variables were collection site (indoor = 1, outdoor = 2), studied neighbourhood and day of survey. For the binary explanatory variables, collection site (indoor = 1, outdoor = 2), indoor was arbitrarily considered as the reference level in the regression analysis. Neighbourhood and day of survey were considered random factors to account for unmeasured variability of mosquito counts, and site was considered fixed factor. All data analysis were performed using the software R v. 4.2.2 ^57^.

### Ethical clearance

This study received ethical approval from the Comité Nacional de Bioética para Saúde de Moçambique (CNBS) of the Ministry of Health of Mozambique (MISAU), reference 208/CNBS/15. Collectors were provided with Fansidar® (Sulfadoxine-Pyrimethamine) to reduce the likelihood of malaria infection or transmission, in accordance with the recommendations of the Mozambique National Malaria Control Program. This type of preventive measure has been shown to reduce the risk of malaria in HLC volunteers by 96.6% in similar studies carried out elsewhere ^58^.

### Consent

Written informed consents were obtained from all individuals that accepted to participate in the study.

## Results

### Relative abundance of nocturnally active anthropophagic mosquitoes

A total of 69,758 host-seeking female mosquitoes belonging to twenty-three species and four genera were caught during 35 nights of paired human landing collections indoor (n=19,752) and outdoor (n= 50,006). The genera *Anopheles*, *Culex* and *Mansonia* were the most common ( Table 1). Among species in the genus *Anopheles*, *An. gambiae s.l* were the most common [63.2% (7,840/12,401)], followed by *An. pharoensis* [16.0% (1,978/12,401)], *An. ziemanni* [11.8% (1,466/12,401)] and *An. tenebrosus* [8.5% (1,048/12,401)]. The genus *Culex* was mostly represented by *Cx. tritaeniorhynchus* [38.5% (8,511/22,078)], followed *Cx. pipiens s.l* [27.1% (5,990/22,078)], *Cx. poicilipes* [25.6% (5,663/22,078)], and *Cx. antennatus* [8.4% (1,854/22,078)]. The genus *Mansonia* comprised *Ma. uniformis* [88.3% (31,068/35,183)] and *Ma. africana* [11.7% (4,115/35,183)]. The genus *Aedes*, comprised three species, of which *Ae.* ( *Coetzeemyia*) *fryeri* was the most predominant species caught [89.5% (77/86)], followed by *Ae*. ( *Muscidus*) *sudanensis* [5.8% (5/86)] and *Ae*. ( *Stegomyia*) *subargenteus* [4.7% (4/86)]. *Coquillettidia aurites* was the most common of the *Coquillettidia* collected ( Table 1).

**Table 1.** Relative abundance of nocturnally active mosquito species collected by human landing catches in Massavasse village, southern Mozambique from February to April 2016.

Species	Indoor	Outdoor	Total
*Anopheles gambiae s.l.*	3403	4437	7840
*Anopheles pharoensis*	28	1950	1978
*Anopheles ziemanni*	53	1413	1466
*Anopheles tenebrosus*	25	1023	1048
*Anopheles funestus*	40	29	69
*Aedes fryeri*	4	73	77
*Aedes subargenteus*	0	4	4
*Aedes sudanensis*	1	4	5
*Coquillettidia aurites*	1	8	9
*Coquillettidia versicolor*	0	1	1
*Culex tritaeniorhynchus*	1007	7504	8511
*Culex pipiens s.l.*	1019	4971	5990
*Culex poicilipes*	1219	4444	5663
*Culex antennatus*	229	1625	1854
*Culex quinquefasciatus*	43	9	52
*Culex bitaeniorhynchus*	2	3	5
*Culex sitiens*	1	2	3
*Mansonia uniformis*	11156	19912	31068
*Mansonia africana*	1521	2594	4115

The results of molecular identification carried on a subsample of 806 and 49 mosquito specimens identified morphologically as members of *An*. *gambiae* complex and *An. funestus* group, respectively are depicted in Table 2. Successful amplification was achieved with 379/806 and 320/806 specimens of *An. gambiae* complex obtained from indoor and outdoor subsamples, respectively. Only 17/49 and 13/49 specimens of *An. funestus* from indoor and outdoor successfully amplified ( Table 2). *Anopheles arabiensis* was the most common member of the *Anopheles gambiae* complex, followed by *An. merus*, *An*. *quadriannulatus*, and *An. gambiae s.s. An. gambiae s.s* was only detected in the indoor collections, but *An. arabiensis* was caught biting indoors and outdoors. Of *An. funestus* group, two species were recorded: *An. funestus s.s* was found in indoor and outdoor samples, whereas *An. parensis* was only found in the indoor catches ( Table 2).

**Table 2.** Result of PCR analyses for molecular identification of sibling species members of *Anopheles gambiae* complex and *An. funestus* group.

Species complex/ group	Molecular ID	Total	
		Indoor	Outdoor
*Anopheles gambiae*	*Anopheles gambiae * *s.s.*	2	0
	*Anopheles* *arabiensis*	334	270
	*Anopheles merus*	40	28
	*Anopheles* *quadriannulatus*	3	22
*Anopheles funestus*	*Anopheles funestus* *s.s.*	10	13
	*Anopheles parensis*	7	0
*Anopheles gambiae*	Not amplified	24	83
*Anopheles funestus*		9	10

### Nocturnal biting rhythms

**Figure 3.**
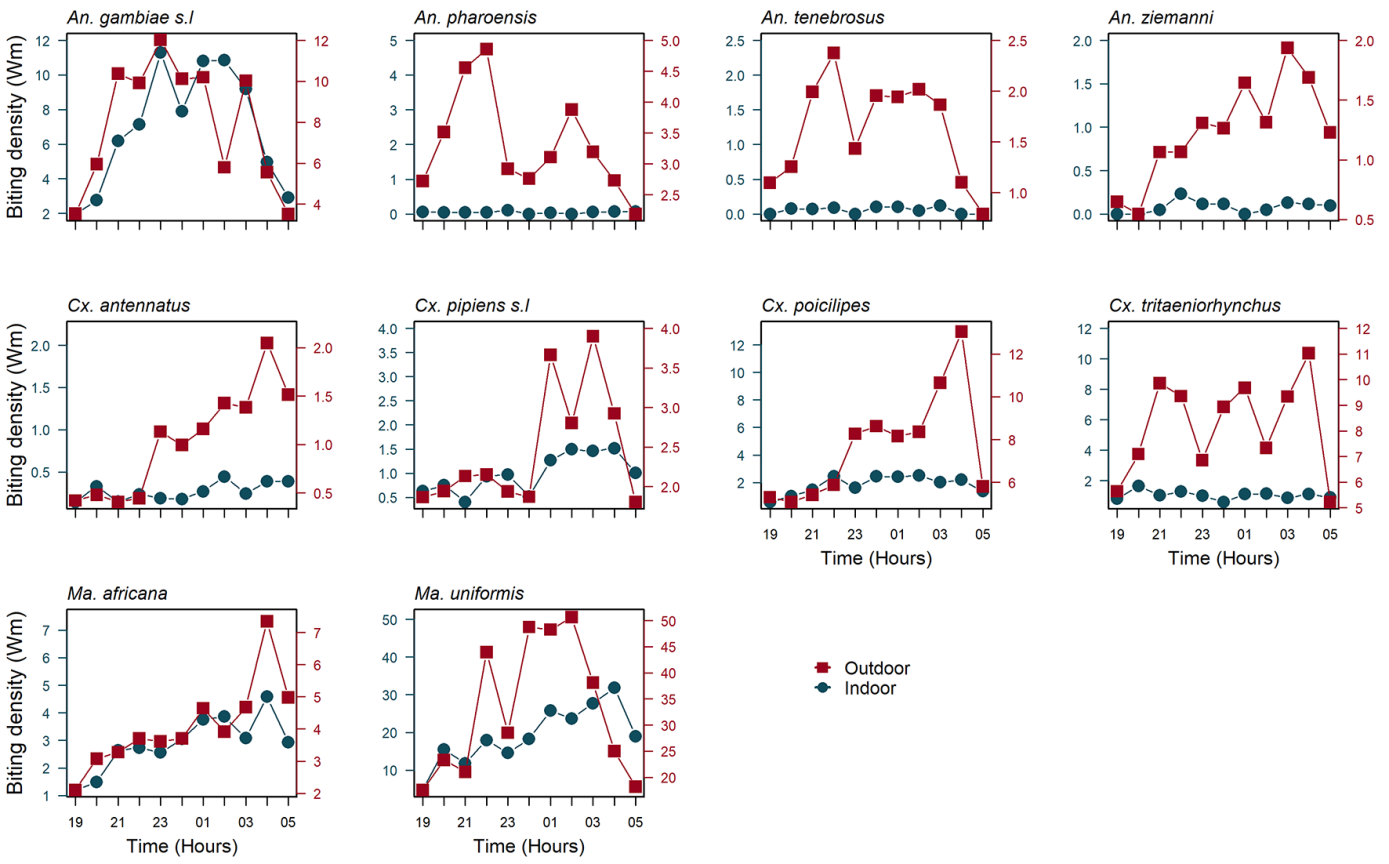
Crude indoor and outdoor hourly (19:00 to 05:00) mean biting density per person/site/night by the predominant mosquito vector species in Massavasse village from February to April 2016. Biting densities were determined by human landing catches and, are expressed in term of Williams mean (Wm) (see methods).

The crude mean biting densities of the most common mosquito species already known to be malaria or arbovirus vectors are depicted in Figure 3. Indoor and outdoor activity of *An. gambiae s.l* showed similar patterns, with the biting peak time ranging from around 22:00 to 03:00. The outdoor biting peak in the biting rhythms of *An. tenebrosus* and *Cx. tritaeniorhynchus* resembled that of *An. gambiae s.l*. Conversely, the outdoor activity of *An. pharoensis* showed a bimodal pattern with two distinct peaks, the larger peak occurring around 22:00, followed by a second small peak in the early morning (02:00). The outdoor biting activity of *An. ziemmani*, *Cx antennatus* and *Cx. poicilipes* peaked later in the morning, around 04:00. These vector species, together with *An. pharoensis*, *An. tenebrosus* and *Cx. tritaeniorhynchus* were actively seeking hosts at a lower intensity indoor throughout the night, but without an obvious peak. Indoor and outdoor biting activity of *Cx. pipiens s.l* and *Ma. africana* peaked around 01:00 to 03:00 ( *Cx. pipiens s.l*) and 04:00 ( *Ma. africana*) ( Figure 3). *Man. uniformis* outdoor biting peaked at 04.00, two hours behind its activity peak indoors ( Figure 3).

Negative binomial regression model analyses indicated that a typical person was likely to be bitten by *An. gambiae s.l* with nearly same intensity indoors and outdoors throughout the night ( Table 3), whereas biting by all other vector species were far more likely to occur outdoors ( Table 3).

**Table 3.** Abundance and crude nocturnal human-biting rates (# bites/person/night) by the predominant mosquito taxa in Massavasse village, February to April 2016.

Species	Total collected		Biting rate *		IRR (± 95%CI) **
	Indoor	Outdoor	Indoor	Outdoor	
*An. gambiae s.l.*	3,403	4,437	87.00	75.99	1.10 (0.97 - 1.26)
*An. pharoensis*	28	1,950	0.54	36.41	3.18 (1.91 - 5.28)
*An. ziemanni*	53	1,413	0.90	13.71	4.89 (2.89 - 8.30)
*An. tenebrosus*	25	1,023	0.06	1.62	4.87 (2.91 - 8.14)
*Cx. tritaeniorhynchus*	1,007	7,504	11.47	90.33	4.33 (3.39 - 5.53)
*Cx. pipiens s.l.*	1,019	4,971	10.96	26.99	2.91 (2.12 - 3.99)
*Cx. poicilipes*	1,219	4,444	20.06	84.58	2.52 (2.12 - 2.99)
*Cx. antennatus*	229	1,625	0.27	1.04	4.82 (3.34 - 6.96)
*Ma. africana*	1,521	2,594	31.89	45.03	1.40 (1.19 - 1.64)
*Ma. uniformis*	11,156	19,912	210.68	363.33	2.05 (1.77 - 2.38)

*Crude biting rate expressed as Williams mean (Wm). **Indoor collections were considered as reference in GLMM models. IRR – Incidence Risk Ratio estimates from negative binomial GLMM models.

**Figure 4.**
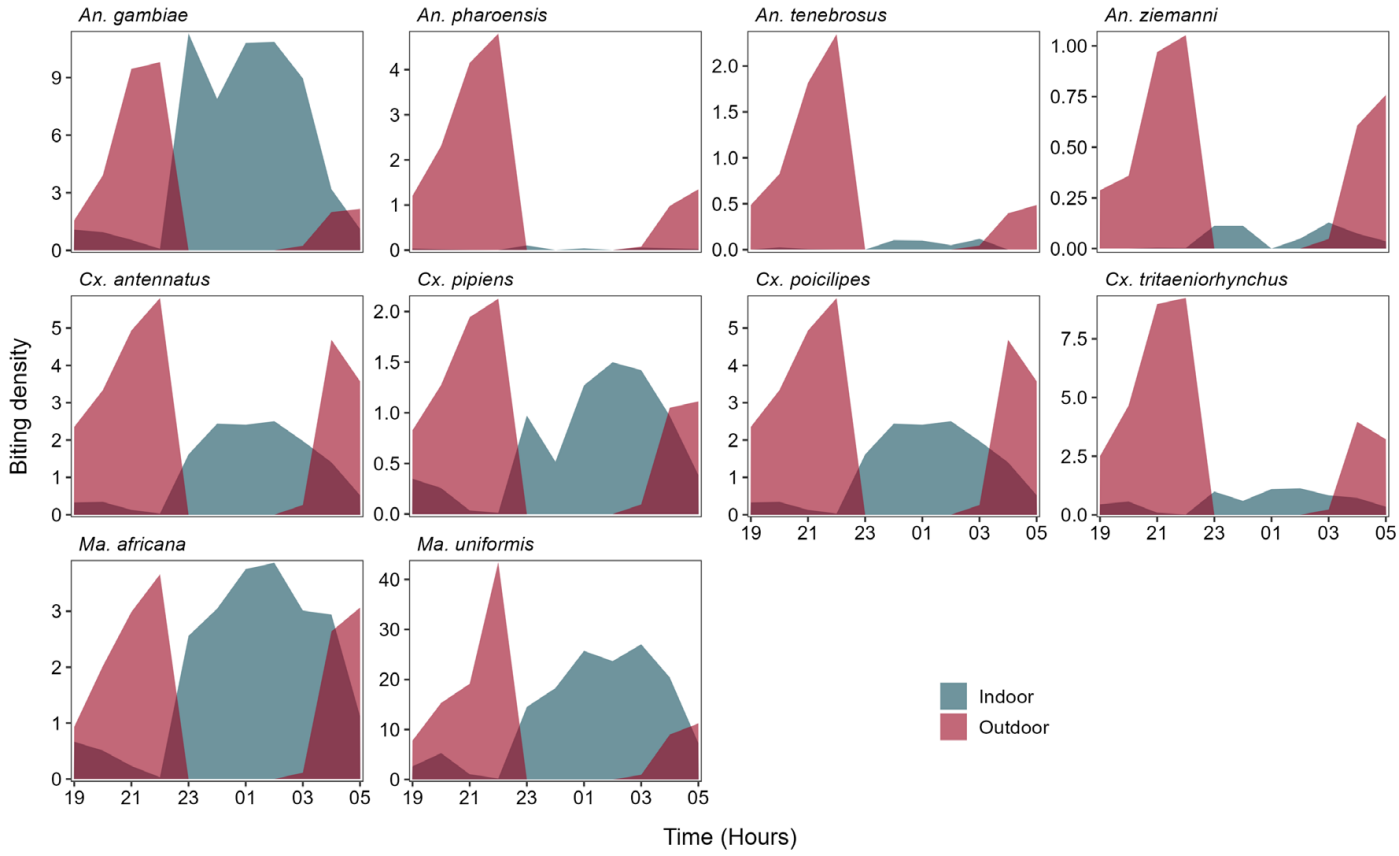
Mean hourly adjusted biting exposure in unprotected individuals by dominant mosquito vector species in Massavasse village. Biting exposure were adjusted by multiplying the crude biting rate by the proportion of people indoor and outdoor at night.

Sleep survey data (underlying data https://doi.org/10.17605/OSF.IO/AYFM6) indicated that all inhabitants typically retired indoors by 22:00 and emerged in the morning from 03:00. Estimates of human-biting exposures, adjusted by multiplying the crude biting rate by the proportion of people indoors and outdoors at night, indicated that an unprotected individual (i.e., a non-user of ITNs) would receive an average of 85.93 bites/night by *An. gambiae s.l*, of which 66% were indoors and 34% outdoors ( Table 4). For an LLIN user, biting exposure would be reduced by 53% *(i.e.,* 40.5 bites/night), assuming an ITN protective efficacy of 80%. Results also indicated that both protected and non-protected individuals were exposed to similar numbers of *An. pharoensis*, *An. tenebrosus*, *An. ziemanni*, and *Cx. tritaeniorhynchus* bites because using an ITN may reduce biting exposure to these vector species by only 2%, 5%, 9% and 14%, respectively ( Table 4). Modest reductions in biting exposure would also be expected for *Cx. pipiens s.l* (38%), *Cx. poicilipes* (28%), *Cx. antennatus* (31%), *Ma. africana* (47%) and *Ma. uniformis* (46%). The dynamics of mosquito biting exposure for non-user individuals are depicted in Figure 4. For nearly all species, a high number of bites on a non-protected individual occurred before bedtime, followed by a small increase of bites in the early morning. Most biting would be expected during late evening before bedtime.

**Table 4.** Estimates of mosquito biting exposure (bites/person/night) in non-protected (non-user and protected (ITN users) individuals in Massavasse village (February to April 2016); exposure values determined by multiplying the crude biting rate by the proportion of people indoors or outdoors at night.

Species	Total biting exposure		Biting reduction (%)	Biting exposure (non-user)		Proportion of bites (non-user)	
	Non-users	ITN-users	ITN-users	Indoor	Outdoor	Indoor	Outdoor
*An. gambiae s.l.*	85.93	40.50	52.87	56.79	29.14	0.66	0.34
*An. pharoensis*	15.20	14.94	1.71	0.33	14.87	0.02	0.98
*An. ziemanni*	4.60	4.19	9.06	0.52	4.08	0.11	0.89
*An. tenebrosus*	6.81	6.48	4.74	0.40	6.40	0.06	0.94
*Cx. tritaeniorhynchus*	39.65	34.17	13.82	6.85	32.80	0.17	0.83
*Cx. pipiens s.l.*	16.12	9.97	38.16	7.69	8.43	0.48	0.52
*Cx. poicilipes*	38.65	27.69	28.36	13.70	24.95	0.35	0.65
*Cx. antennatus*	4.94	3.40	31.19	1.93	3.01	0.39	0.61
*Ma. africana*	37.16	19.77	46.81	21.75	15.42	0.59	0.41
*Ma. uniformis*	252.96	136.06	46.21	146.13	106.83	0.58	0.42

## Discussion

Understanding the behavioural preferences and habits of local populations of disease vectors is crucial for optimising control measures and enhancing epidemic preparedness against emergent mosquito-borne diseases. Although a wealth of studies exist on the malaria vectors of the region ^31, 59^, this study is the first to provide a description of the nocturnal biting rhythm of *Anopheles* mosquitoes frequently found in the malaria - endemic area around Massavasse village and to provide direct measures of human exposure to biting by arbovirus vector species that are frequently found in rural settings of Mozambique ^21^.

Biting activity increased in all mosquito species during early evening. In *An. gambiae s.l.,* biting activity extended from early evening to early morning, biting indoors and outdoors at nearly the same intensity. This pattern of *An. gambiae s.l* biting activity differed from other studies which reported peak activity at around 04:00 - 06:00 ^33, 60– 65^. However, the discrepancy may be explained by the fact that the vector population in the present study was not homogeneous. Molecular analyses confirmed the presence of *An. arabiensis*, *An. merus* and *An. quadriannulatus* and *An. gambiae s.s*, in descending order of abundance. *An. arabiensis*, *An. merus* and *An. quadriannulatus* are well known exophagic malaria vectors which can blood-feed both indoors and outdoors. *An. gambiae s.s*, on the other hand, is a strongly endophilic vector ^66^. In fact, the biting pattern exhibited by *An*. *gambiae s.l* in this study resembles that of *An*. *arabiensis* reported in Matola city, also in southern Mozambique ^32^, and by *An. merus* in Kenya ^67, 68^. Marked spatial and temporal variation in host-seeking rhythms among members of the *An. gambiae* complex have also been reported elsewhere ^33^. The variabilities may reflect geographic differences in environmental factors modulating host-seeking activity ^37, 69– 71^, or the eco-evolutionary and behavioural adaptation of mosquito vectors to the human population’s habits, including the control measures used by different host populations. Evidence that shifts in mosquito behavioural preferences can occur following introduction of insecticide-based control interventions comes from studies on *An. farauti* in Solomon Island ^37^, *An. funestus* in Benin ^38, 72^, and *An. gambiae* in Burkina Faso and Equatorial Guinea ^73, 74^. All have reported changes from indoor feeding behaviour to a predominantly exophagic habit following the intense insecticide pressure of scaled up indoor residual spraying and ITN coverage.

Host-seeking activity of *An. pharoensis* occurred almost entirely outdoors. The bimodal biting rhythm of *An. pharoensis* reported here, with a peak of activity around 22:00 hrs and a smaller peak around 02:00in the morning, was similar to that described by Haddow ^61^ and Chandler ^64^, except that in their study the largest peak was the latter one. Indoors, *An. pharoensis* bit at a steady low rate throughout the night. This pattern differed from the unimodal rhythm reported in Burkina Faso ^62^, and Kenya ^75^, where peak activity occurred from 21:00 - 23:00. However, in the Kano plain, also in Kenya, Chandler and colleagues ^64^ reported a peak around 05:00-06:00in the morning. These data show that differences in nocturnal host-seeking peaks of activity also exist in *An. pharoensis*, underscoring the need for a thorough investigation of this vector species for implementation of site appropriate malaria control interventions. *An. pharoensis* is an important vector of malaria and lymphatic filariasis across its range of occurrence ^43, 76^. However, information on the bionomics of this vector is poor for most of Africa, as is knowledge of its role as a malaria vector. The high level of biting recorded when people were active outdoor suggests that, while it is normally only a secondary vector *An. pharoensis* could play an far greater vectorial role in maintaining outdoor residual malaria transmission in Massavasse village following scale-up of indoor-targeted control.

*Anopheles tenebrosus* and *An. ziemanni,* were the two most abundant sibling species of the *An. coustani* group, and both showed different biting patterns. Host-searching activity was concentrated outdoor for both species, but while activity of *An. tenebrosus* extended from 21:00–03:00, *An. ziemann*i activity increased gradually peaking around early morning. An irregular biting rhythm of a population of *An. ziemanni* was reported by Haddow ^52^ in Uganda. Published studies on the biting behaviour of *An. tenebrosus* and *An. ziemanni* and, as well as their contribution to residual malaria transmission are few with most reports describing the biting behaviour of the *An. coustani* group, which is unlikely to be of much use, in the light of our study data. With recent evidence showing that the two species might be vectors of secondary importance in Tanzania ^77^ and Cameroon ^78, 79^ there is renewed interest. The presence of these vector species in Massavasse village is a concern, as they could contribute to the persistence of malaria in the region. Moreover, they should not be overlooked as part of the ongoing cross boarder malaria elimination initiative involving Mozambique, South Africa, and Swaziland (MOSASWA).

To our knowledge, there are no recent published reports describing the patterns of human exposure to bites by culicine mosquitoes in Africa, though many are known to be vectors of arboviruses and of global or regional medical importance. The recent global expansion of *Aedes aegypti,* together with the relentless urbanisation trends of human populations have led to increased contact between this vector and humans resulting in more frequent and larger outbreaks of dengue, chikungunya and Zika, threatening vast human populations worldwide. While this vector is of major concern, it must not distract from the very real threat to rural or peri-urban human populations, associated with the presence of so many nocturnally active vector mosquitoes.

The present and earlier surveys conducted at the Massavasse site ^31^ confirm the presence of a diverse and range of abundant culicine mosquitoes, notably *Cx. tritaeniorhynchus*, *Cx. antennatus*, *Cx. poicilipes*, and *Cx. pipiens s.l*, *Ma. africana* and *Ma. uniformis*. Some are known to be vectors of important arboviral diseases. *Cx. tritaeniorhynchus* and *Cx. antennatus* are two important vectors of Japanese Encephalitis virus and Rift Valley fever virus in Asia ^80^ and Africa ^47, 81^, respectively. *Cx. poicilipes* and *Cx. pipiens s.l* are important vectors of West Nile fever virus ^82– 84^, while *Ma. africana* and *Ma. uniforms* have been incriminated as vectors of lymphatic filariasis in Ghana ^85^. The transmission risk of both Rift valley fever and lymphatic filariasis is high in the central and northern regions of Mozambique ^12, 86, 87^. Given the high risk of miscarriage in pregnant women infected with Rift valley fever virus ^88^, a thorough study to identify the actual vector species among the potential vectors present is an essential preliminary step before planning measures to mitigate RVF outbreaks. In this study, we found that *Cx. tritaeniorhynchus* were essentially exophagic, biting outdoors at a relatively high intensity from early evening through to the following morning before sunrise. In contrast, indoor activity was consistently low. The pattern of biting rhythm observed here is inconsistent with the bimodal biting rhythm commonly reported in Asia ^89– 92^. Yajima *et al.*, ^93^ have argued that the bimodal rhythm in *Cx*. *tritaeniorhynchus* population is related to variations in physiological age in the host-seeking populations, with a higher proportion of younger females biting earlier at the evening than the older females, which would usually bite later in the night ^93^. Other authors also suggested that variations of environmental temperature and humidity may also have an important role ^90^. So far, we have not found a published report on the biting behaviour of an African population of *Cx*. *tritaeniorhynchus*. The knowledge gap may probably be driven by the fact that the species does not appear to have yet been incriminated as a vector of any human or mammalian pathogen in the African continent. However, the large numbers of host-seeking *Cx*. *tritaeniorhynchus* collected in Massavasse villag is a matter of concern and should stimulate re-evaluation of health system preparedness for possible outbreaks of *Cx*. *tritaeniorhynchus*-transmnitted pathogens in the region. Moreover, the rapid widespread demographic transformation, characterized by increasing mobility and connectivity between Japanese encephalitis virus (JEV) endemic regions in Asia and African may exacerbate the risk of JEV outbreaks in the African continent, where the vector species is also endemic.

As with *Cx. tritaeniorhynchus*, *Cx*. *antennatus* was also highly aggressive outdoors, with a unimodal pattern of activity and a notable peak in the morning. A not dissimilar unimodal pattern, but with the peak in activity occurring around 21:00-23:00, was reported in Kenya ^94^. The discrepancies are likely to be driven by the variability between hosts and vector habitats between the two geographic regions, ecological adaptations or in the type of sampling methods employed, in this study (HLC) and in the Kenyan study (baited net trap).

The remaining vector species, *Cx. poicilipes*, *Cx. pipiens s.l*, *Ma. africana*, and *Ma. uniformis* displayed rather similar unimodal intense patterns of activity both indoor and outdoor, usually peaking from dawn to late morning (01:00 - 04:00). The biting patterns of *Cx*. *poicilipes*, *Ma*. *africana* and *Ma*. *uniforms* were similar to those reported previously in studies from other geographic locations ^52, 94– 96^. The biting activity of *Cx. pipiens s.l* was similar to that reported in west and east Africa for one of the sibling species of the *Cx. pipiens* group, *Cx. quinquefasciatus* ^95, 97^. The estimates of mean biting exposures adjusted for local people nocturnal habits corroborate the crude biting exposures estimates indicating that the main “hotspot” of high biting exposure by both malaria and arboviral disease mosquito vectors in Massavasse village is outdoor before bedtime. For some members of the *An. gambiae* complex found in the village, possibly *An. arabiensis*, and non-Anophelines such as *Cx. pipiens s.l. Cx. poicilipes*, *Ma. africana* and *Ma. uniformis*, a great exposure to bites may also occur indoor throughout bedtime, and outdoor before sunrises. The answers to queries on sleeping habits indicated that residents would retire indoor early in the evening to sleep but emerge early in the morning before sunrise. This pattern of sleeping behaviour is common in agricultural and livestock producing areas where people tend to rise earlier to take care of livestock and work on the land before it becomes too hot. Indoor residual spraying (IRS) and long-lasting insecticide treated (LLINs) spearhead vector control interventions worldwide ^23^. However, the efficacy of both methods depends on the propensity of the vectors to feed and rest inside human shelters or houses ^98^. Our findings indicate that malaria and arbovirus vectors in Massavasse village may be beyond the reach of IRS and ITN interventions, given the high tendency to outdoor feeding shown by the local mosquitoes. The findings emphasize the importance of baseline study of the target vector population before selecting control methods, and ensuring that all methods integrate fully when implemented, with each other and with other public health interventions. The study also highlights the urgent need for novel effective control tools that aer effective against outdoor biting mosquitoes whose vectorial role includes malaria and numerous arboviruses.

## Abbreviation

HBR: Human biting rate

HLC: Human-landing Catch

ITN: Insecticide Treated Net

MBD: Mosquito-borne disease

PCR: Polymerase Chain reaction

## Data Availability

Open Science Framework: Characterisation of human exposure to nocturnal biting by malaria and arbovirus vectors in a rural community in Chókwè district, southern Mozambique: https://doi.org/10.17605/OSF.IO/AYFM6 ^99^ This project contains the following underlying data: Dataset_1: Raw dataset of mosquito species collected using human-landing catch approach in Massavasse village from February to April 2016 Dataset_2: Raw dataset of sleeping and waking time of people in Massavasse village from February to April 2016 Open Science Framework: Characterisation of human exposure to nocturnal biting by malaria and arbovirus vectors in a rural community in Chókwè district, southern Mozambique: https://doi.org/10.17605/OSF.IO/AYFM6 ^99^ This project contains the following extended data: - Questionnaire_1: A copy of the questionnaire used to investigate the sleeping habits of Massavasse village residents Data are available under the terms of the Creative Commons Zero “No rights reserved” data waiver (CC0 1.0 Public domain dedication).
