# Supplementary material for: Characterisation of human exposure to nocturnal biting by malaria and arbovirus vectors in a rural community in Chókwè district, southern Mozambique
Source: Wellcome Open Res. 2023 May 2;8:193. [Version 1] doi: 10.12688/wellcomeopenres.19278.1 (PMC10357080; doi:10.12688/wellcomeopenres.19278.1)
Supplement: Peer review history

# Reviewer report: Reviewer response for version 1

**DOI:** 10.21956/wellcomeopenres.21361.r62978

**Published:** 2023-09-07

**Authors:** John B Keven

**Affiliations:** 1 Department of Population Health and Disease Prevention, University of California Irvine, Irvine, California, USA

This work is generally sound, but I have a few comments and suggestions, all minor issues that can be easily addressed by the authors.

**Methods**

*Paragraph 1, line 6*: "a well characterized mosquito population".

*Comment*: "Community" is a more appropriate word to use as it encompasses all mosquito species in the area. "Population" is used to refer to one species only.

*Paragraph 4, line 10*: "participate".

*Comment*: Did you mean "participant"?.

*Paragraph 8 (Estimation of Human Biting Rates), line 3*: "greater than 1% of all mosquitoes".

*Comment*: Why 1% and not another value? Please explain the reason for setting this criteria at 1%.

 Paragraph 13, line 7: "level of 80% (P = 0.8) for ITNs".

*Comment*: The P = 0.8 can be confused for a P value associated with a statistical significance test.

**Results**

*Paragraph 1, subtitle*: "Relative abundance of nocturnally active anthropophagic mosquitoes".

*Comment*: I think that "anthropophilic" is a more appropriate term to use in this context than "anthropoophagic". Anthropohagic specifically means that the mosquitoes had fed on human blood, which is not the case here because these mosquitoes were unfed ones.

*Paragraph 1, line 3-4*: "paired human landing collections indoor (n=19,752) and outdoor (n= 50,006)"

*Comment*: By "paired", do you mean that mosquitoes were collected outside and inside a house simultaneously (i.e., at the same time)? If so, then your results might be biased by superficially showing a higher outdoor biting rate relative to indoor biting rate. This is because as host-seeking mosquitoes commute from peridomestic habitats towards houses to seek human hosts, most of the mosquitoes that would have otherwise entered into the house (indoor) opportunistically encounter humans outdoor, preventing them from going indoor, causing a superficially high number of outdoor compared to indoor biting rate. An unbiased sampling approach would be to conduct one sampling method (indoor or outdoor but not both) per house but not simultaneously. Please address this potential caveat.

**Discussion**

*Paragraph 2, line 6-11*: "However, the discrepancy

 may be explained by the fact that the vector population

 in the present study was not homogeneous. Molecular analyses confirmed the presence of An. arabiensis, An. merus and An. quadriannulatus and An. gambiae s.s, in descending order of abundance".

*Comment*:  Why not produce biting activity profile for each species separately, or at least the dominant species, e.g. An. arabiensis? This will help solve the discrepancy.

*Paragraph 2, line 12-14*: "An. arabiensis, An. merus and An. quadriannulatus are well known exophagic malaria vectors which can blood-feed both indoors and outdoors."

*Comment*: Contradictory statement. An "exophagic" mosquito species feeds predominantly outdoors not both indoors and outdoors as stated here. Did you mean to say exophilic? If so, then this may solve the contradiction because exophilic means a different thing from exophagic.

Is the work clearly and accurately presented and does it cite the current literature?

Yes

If applicable, is the statistical analysis and its interpretation appropriate?

Yes

Are all the source data underlying the results available to ensure full reproducibility?

Yes

Is the study design appropriate and is the work technically sound?

Partly

Are the conclusions drawn adequately supported by the results?

Yes

Are sufficient details of methods and analysis provided to allow replication by others?

Partly

Reviewer Expertise:

Medical entomology, vector biology, molecular biology

I confirm that I have read this submission and believe that I have an appropriate level of expertise to confirm that it is of an acceptable scientific standard, however I have significant reservations, as outlined above.

# Reviewer report: Reviewer response for version 1

**DOI:** 10.21956/wellcomeopenres.21361.r57228

**Published:** 2023-07-20

**Authors:** Gerry F Killeen

**Affiliations:** 1 School of Biological, Earth & Environmental Sciences and Environmental Research Institute, University College Cork, Cork, County Cork, Ireland

Generally speaking, this is a nice, well thought-through and well-implemented study, which has been nicely written up into a smooth-flowing, well-crafted manuscript. Having said that, the are several substantive opportunities to improve the technical rigour of the estimations and statistical contrasts, as well as the transparency and presentation formats for the results, all of which I detail as follows.

1. While it’s nice to see our old paper from 2006 being used and cited, we have recently published two more suitable manuscripts summarizing the most up-to-date and carefully annotated versions of these analytical models. I recommend the authors use these latest papers, which capture all the various iterative stages of evolution of these approaches to calculating and graphing exposure patterns, and include lots of useful templates in the supplementary materials:
  Monroe et al 2020 Malaria Journal 19: 207; https://malariajournal.biomedcentral.com/articles/10.1186/s12936-020-03271-z ^1^
  Killeen et al 2021 Parasites & Vectors 13: 384; https://parasitesandvectors.biomedcentral.com/articles/10.1186/s13071-021-04884-2 ^2^
2. Figure 3 also needs the human sleeping pattern data included, so that readers can see how, where and when it coincides with mosquito activity, to yield figure 4.
3. I would strongly prefer a stacked graph format for figure 4, which is otherwise quite good, as per the examples provided above and below.
4. A Williams’ mean is inappropriate as it underrepresents the small proportion of nights and locations during which the majority of actual exposure occurs: while log transformations are great for making valid statistical comparisons for over dispersed data, they inaccurately dampen down the important contributions of the highest density and articially smoothen off activity profiles that are naturally every bit as dynamic as the raw data and simple arithmetic means indicate. Please use simple arithmetic means instead, which may well yield somewhat higher but more accurate estimates for the proportions of exposure occurring while asleep and preventable by LLINs for both *An. gambiae* and *Cx. quinquefasciatus*.
5. For simple ways to analyze mosquito preferences for feeding indoors versus outdoors, during sleeping versus waking hours, or the interaction between the two, logistic regression with simple binomial distributions for binary format expression of where and when mosquitoes are captured is far more appropriate and powerful than Poisson or Negative Binomial regression. Please see the second paper above, plus the following example of a practical application, for details: Huho et al Int J Epidemiol 42: 235; https://academic.oup.com/ije/article/42/1/235/698545 ^3^

Is the work clearly and accurately presented and does it cite the current literature?

Partly

If applicable, is the statistical analysis and its interpretation appropriate?

Partly

Are all the source data underlying the results available to ensure full reproducibility?

Yes

Is the study design appropriate and is the work technically sound?

Partly

Are the conclusions drawn adequately supported by the results?

Partly

Are sufficient details of methods and analysis provided to allow replication by others?

Yes

Reviewer Expertise:

Malaria vector biology and control

I confirm that I have read this submission and believe that I have an appropriate level of expertise to confirm that it is of an acceptable scientific standard, however I have significant reservations, as outlined above.

## References

[1] : Methods and indicators for measuring patterns of human exposure to malaria vectors. *Malar J* .2020;19(1) : 10.1186/s12936-020-03271-z 207 10.1186/s12936-020-03271-z 32546166PMC7296719

[2] : Simplified binomial estimation of human malaria transmission exposure distributions based on hard classification of where and when mosquitoes are caught: statistical applications with off-the-shelf tools. *Parasit Vectors* .2021;14(1) : 10.1186/s13071-021-04884-2 384 10.1186/s13071-021-04884-2 34344438PMC8336365

[3] : Consistently high estimates for the proportion of human exposure to malaria vector populations occurring indoors in rural Africa. *Int J Epidemiol* .2013;42(1) : 10.1093/ije/dys214 235-47 10.1093/ije/dys214 23396849PMC3600624

# Reviewer report: Reviewer response for version 1

**DOI:** 10.21956/wellcomeopenres.21361.r59350

**Published:** 2023-07-19

**Authors:** Dickson Wilson Lwetoijera

**Affiliations:** 1 Environmental Health and Ecological Science Department, Ifakara Health Institute, Ifakara, France

This study summarizes important findings on how biting risk of different malaria and non-malaria disease mosquitoes resonate with human sleeping patterns. Although with notable limitations, the article highlights the importance of analyzing mosquitoes biting patterns by including secondary malaria vectors and arboviruses transmitting mosquitoes even in locations that are well covered with bednets, and its contribution to outdoor and residual malaria transmission.

1. This sampling period is also characterized by high densities of mosquitoes in the area, and one would expect the actual risk to be estimated from proportion of infectious bites out of all received bites either by protected or unprotected individual, which isn't the case. This represents a major limitation of this study.
2. Another limitation is that the collection were done in a simulated house environment (experimental hut) that by far differs from an actual house which would have had concentrated indoor host’s air plume differently to host seeking mosquitoes and probably caused variability in the recorded numbers.
3. At a sentence “ *Paired indoor and outdoor human landing catches were conducted in neighbourhoods two to six of Massavasse village*” it is a little confusing, did the author meant to say that collections were conducted in six neighbourhood of Massavasse village?
4. In the methodology, it reads as collections were done for only six hours, given that collectors had to break for every following our, but in the biting patterns graph, it looks like there is 11 collection points. Can authors clarify on this.
5. Given that this was done in the field, with history of high malaria prevalence, authors didn’t clarify if the mosquito collectors were protected with malaria prophylaxis or not, even if they were fully trained and experienced mosquito collectors.
6. Authors can also fix minor typos, such as “aer” to be replace with “are”; villag with village, bit with bite; *Cx. Antennatus *with *Cx. antennatus*

Is the work clearly and accurately presented and does it cite the current literature?

Yes

If applicable, is the statistical analysis and its interpretation appropriate?

Yes

Are all the source data underlying the results available to ensure full reproducibility?

Yes

Is the study design appropriate and is the work technically sound?

Yes

Are the conclusions drawn adequately supported by the results?

Yes

Are sufficient details of methods and analysis provided to allow replication by others?

Yes

Reviewer Expertise:

Medical entomologist, specialized in malaria vectors behaviors and its interaction to control interventions.

I confirm that I have read this submission and believe that I have an appropriate level of expertise to confirm that it is of an acceptable scientific standard, however I have significant reservations, as outlined above.

# Reviewer report: Reviewer response for version 1

**DOI:** 10.21956/wellcomeopenres.21361.r59342

**Published:** 2023-07-19

**Authors:** Patric Stephane Epopa

**Affiliations:** 1 Direction Régionale de l'Ouest, Institut de Recherche en Sciences de la Santé, Bobo-Dioulasso, Burkina Faso, South Africa

In the study entitled: “ **Characterisation of human exposure to nocturnal biting by malaria and arbovirus vectors in a rural community in Chókwè district, southern Mozambique**” the authors describe the night biting patterns of all mosquitoes present in the village of Massavasse in southern Mozambique during a 3 months period of rainy season 2016 (February to April 2016). Results indicates significant nocturnal biting activities from almost all the species found. Outdoor biting activities was in general very high, not only for malaria mosquitoes but also for other Culicidae of public health importance, which can act as vectors for some important arboviral disease (such as dengue, West Nile fever, Japanese Encephalitis virus or Rift Valley fever ). The authors use these results confirm the threat of malaria mosquito vectors in the village but also to highlight the increasing importance of the other mosquito vectors and the potential risk that these could have in generating unexpected outbreaks if nothing is done.

 In general, the manuscript is well written and most of the time easy to understand. The language is simple with a quite good English. Nevertheless, a number of edits are still needed in my opinion to improve the understanding and bring some details necessary to ensure study reproduction.

**The abstract** is generally well presented, with nevertheless some minor edits needed to improve understanding of the study (see detailed comments below).

**The introduction** section is globally well written. The context and aims of the study are quite well described. Nevertheless, some minor edits will be needed to improve understanding by the readers (see detailed comments below). I will also recommend adding one or two sentences to explain a little bit more the rationale behind the study for this is a bit confusing in the present manuscript. Is the study tries to describe the dynamic of all mosquitoes of health importance in the village to highlight the need of more structure surveys at country level ? or rather aim to give a background data to support future control interventions ? etc… This really needs to be clarified.

**The Methods** section quality is just average. Though generally well written with a simple language and a quite good level of details in general, some important details are lacking and will needed completion to ensure study reproducibility. In addition, some aspects of the study design are confusing. Also, some information given in the figures illustrating the Methods section do not match with the text in the manuscript. Nevertheless, almost all of these lacks will just require minor edits in general. I strongly recommend the authors to address those issues in the revised version of the manuscript to ease understanding of their study and its reproducibility.

**The results** section is very well structured, and results presented in a simple way easy to understand and generally well-illustrated. The tables and figures are in general of good quality. Only minor edits are needed here to improve readership.

**The Discussion** section is also of good quality. The references and comparisons given are generally appropriated. The only thing that is lacking is a description of limitations of the study.

**The conclusion** section is also well written. Conclusions given are in general appropriate even though some of them will need to be moderated.

**Abstract (pdf version)**

**Background:**

- Line 2 : “ *is fundamental to reduce…*” I will suggest changing to “is fundamental to control…”

** Methods:**

- The period of survey (period of the year) and the collection duration is lacking in the abstract, this is very important for the understanding of your results, I recommend adding them. 

**Results:**

- Just after given the general number of host-seeking female mosquitoes, it could be interesting to add even in brackets the general overall mean exposure per night)

** Conclusion**

- “ *identify a wide range of arbovirus vectors nocturnally active in rural areas,* *many with outbreak potential*” In my opinion, this conclusion is a little bit exaggerated. Unless you add evidence that the corresponding pathogen are already circulating the studied area, there is no real potential of outbreak. So, I will suggest that you moderate your conclusions here. For example, you may say something like: “identify a wide range of arbovirus vectors nocturnally active in rural areas, many of these could present interest in term of public health”

**Introduction (pdf version)**

1. Please update your WHO reference and corresponding statistics whenever possible to the newest malaria report 2022.
2. Paragraph 3: I will recommend to moderate a little bit your conclusions about health authorities disease control policies. I do understand the frustration pf the authors about decision from Mozambican health authorities to allocate resources mainly to malaria control. In fact, I am not really sure that their willing is to consciously neglect the other vector borne disease. Resources for disease control in a low-income country like Mozambique is usually very limited. So the way they are allocated is a result of complex considerations sometimes out of authorities control. This usually aim to optimize the cost/benefits ratio and gives priority to the most urgent needs.
3. Paragraph 5: Line 4-7: “ *Effective vaccines are few, and vector control interventions remain the most reliable and cost-effective control measures against virtually all known **mosquito-borne arboviruses***”
  I think “ **arboviral diseases”** rather than **“mosquito-borne arboviruses”** is more appropriated.
  Line 7: please delete “ **Therefore**.”
4. Line 13: “ *HBR gives the most realistic estimation of the magnitude of human exposure **to a mosquito vector** over time and space*” I will suggest to say: “HBR gives the most realistic estimation of the magnitude of human exposure **to given host seeking mosquito vector** over time and space”

**Methods**

**Mosquito collection**

 This is no very ease to follow and there are lots of details that are not clear at all or even confusing:

- The rotation design: Does it follow a particular scheme (like latin square)? or just random distribution of collector each time?
- What was the minimum distance between the sampling areas?
- How many collectors per day of sampling?
- What do the sampling locations refer to? is it a compound? a house? and what is the correspondence with the “sampling sites” indicated in Figure1?
- Are the terms “sampling sites”, sampling locations and sampling areas referred to the same thing? If yes, you better choose only one expression, if not you should define each in the manuscript to avoid confusion
- In figure 1, 6 sampling sites are indicated. This number do not correspond to nothing (nor the number of neighbourhoods studied, nor the sampling location of the manuscript, nor the number of huts. This will need additional edits from the authors.
- Line 6: “ *a well characterized mosquito population*” do you mean “a well characterized malaria mosquito population”? If so, please correct accordingly.
- Line 12: I will suggest to keep the term ‘cold/dry” as previously stated rather than “winter/dry”
- Line 13: if available, add the confidence interval to the mean rainfall 

**Sleeps surveys**

 Additional information needs to be provided here

- Statement on how you managed individual data privacy/protection
- Inclusion/exclusion critaria
- Compound selection mode (how compounds were selected (systematic? Randomly?)
- Why this sample size? (was this related to a particular expected ratio of compounds? or may be just to the number of acceptance received)
- To ease reader understanding, I will suggest that you start by given a general overview of you study design before going into detailed description. You can also add a small diagram to illustrate your sampling design.

**Data analysis**

- Estimation of human-biting rates: “ *Estimation of human exposure to mosquito bites was only carried out for species where the total catch was equal to or greater than 1% of all mosquitoes caught*” is there a particular reason for that ? please add a rationale to explain a little bit more for the context may be different for other similar study.
- Please be consistent with the mosquito collecting period: in the mosquito collection section it is mentioned 19h00 to 05h00 and in the Data analysis section it is mentioned 19h00 to 06h00.

**Consent**

- I will suggest that you add 2 or 3 sentences to explain a little more about how the information was given and the consent form were taken: by who (field team, specialized stakeholder engagement team); what type of information given (study objectives, risks, etc.), form of signature (formal signature, finger print) 

**Results**

1. I suggest you to split the total number of Anopheles (806) into 2 indicating the respective number of mosquitoes selected indoor and outdoor and for each the number of successful amplification. The same comment applies to Anopheles funestus PCR results.
2. Page 7, Paragraph 2: “ *whereas biting by all other vector species were far more likely to occur outdoors*” I will suggest rephrasing for this gives the impression that indoor collections are negligible. You can say something like: “whereas biting by all other vector species were far more likely to occur outdoors despite significant indoor biting.”
3. Table 3: I suggest adding a column next to the mosquito species to indicate for each the main transmitted diseases (malaria, arboviral diseases). This will highlight the importance of your work in public health perspectives.

**Discussion**

 The author used the density index of vector to indicate the potential risk of disease occurrence in the studied area. The results are used to highlight the potential risk of arboviral diseases outbreak in the area due to significant presence of related biting mosquito vectors.

 Nevertheless, unless the presence of such pathogen has been observed in this area, it is unlikely in my opinion to see such event (vector borne disease being the result of contact between a host, a pathogen and his vector0 for vector and host only are not enough.

Is the work clearly and accurately presented and does it cite the current literature?

Yes

If applicable, is the statistical analysis and its interpretation appropriate?

I cannot comment. A qualified statistician is required.

Are all the source data underlying the results available to ensure full reproducibility?

Yes

Is the study design appropriate and is the work technically sound?

Yes

Are the conclusions drawn adequately supported by the results?

Partly

Are sufficient details of methods and analysis provided to allow replication by others?

Partly

Reviewer Expertise:

medical entomology, malaria vector control, malaria mosquito ecology and biology

I confirm that I have read this submission and believe that I have an appropriate level of expertise to confirm that it is of an acceptable scientific standard, however I have significant reservations, as outlined above.

# Reviewer report: Reviewer response for version 1

**DOI:** 10.21956/wellcomeopenres.21361.r59347

**Published:** 2023-06-29

**Authors:** Yeromin P Mlacha

**Affiliations:** 1 Environmental Health and Ecological Science, Ifakara Health Institute, Ifakara, Morogoro Region, Tanzania

The study's findings have significant implications for understanding the entomological drivers of persistent malaria transmission and the potential for arbovirus outbreaks in Mozambique.

 The paper is well-written, and its findings are succinct and straightforward. The methodology is sound and makes use of tried-and-true methods for collecting and analyzing mosquitoes. Although HLC raises ethical concerns because it exposes the person performing the operation, its usage at hourly intervals enables a thorough assessment of the host-seeking mosquito composition throughout the night.

 The discovery of several nocturnally active arbovirus vectors highlights the necessity of setting up a surveillance system to keep track of these vectors across the country.

 However, It is important to consider the limitations of the study. It would be beneficial to include information on the generalizability of the findings beyond Massavasse village. The discussion and conclusions section may have benefited from a brief paragraph examining limitation and potential solutions.

 Furthermore, the study could benefit from additional discussion on the implications of the findings for vector control strategies. How can the information on biting patterns and the effectiveness of bed nets be used to improve vector control interventions and reduce disease transmission in Mozambique?

 Overall, this study significantly contributes to the understanding of human exposure to mosquito biting and the identification of key vector species in a rural area of Mozambique.

 With some minor additions, this manuscript would be a valuable addition to the journal, providing cohesive insights into mosquito behavior and offering important considerations for vector control efforts.

Is the work clearly and accurately presented and does it cite the current literature?

Yes

If applicable, is the statistical analysis and its interpretation appropriate?

Yes

Are all the source data underlying the results available to ensure full reproducibility?

Yes

Is the study design appropriate and is the work technically sound?

Yes

Are the conclusions drawn adequately supported by the results?

Yes

Are sufficient details of methods and analysis provided to allow replication by others?

Yes

Reviewer Expertise:

Vector-borne diseases

I confirm that I have read this submission and believe that I have an appropriate level of expertise to confirm that it is of an acceptable scientific standard.
